# Impact of early life exposures to geohelminth infections on the development of vaccine immunity, allergic sensitization, and allergic inflammatory diseases in children living in tropical Ecuador: the ECUAVIDA birth cohort study

**DOI:** 10.1186/1471-2334-11-184

**Published:** 2011-06-29

**Authors:** Philip J Cooper, Martha E Chico, Irene Guadalupe, Carlos A Sandoval, Edward Mitre, Thomas AE Platts-Mills, Mauricio L Barreto, Laura C Rodrigues, David P Strachan, George E Griffin

**Affiliations:** 1 Molecular and Biochemical Parasitology, Liverpool School of Tropical Medicine, Pembroke Place, Liverpool, L3 5QA, UK; 2 Laboratorio de Investigaciones FEPIS, Gaspar de Villaroel E8-25 y Seymour, Quito, Ecuador; 3 Laboratorio de Biología Molecular, Hospital de Los Valles, Avenida Interoceánica Km, 12.5, Cumbayá, Quito, Ecuador; 4 Colegio de Ciencias de la Salud, Universidad San Francisco de Quito, Avenida Interoceánica Km, 12.5, Cumbayá, Quito, Ecuador; 5 Department of Microbiology and Tropical Medicine, Uniformed Services University of Health Sciences, 4301 Jones Bridge Road, Bethesda, Maryland, 20814, USA; 6 Division of Allergy and Clinical Immunology, University of Virginia Health System, 1215 Lee Street, Charlottesville, Virginia, 22908, USA; 7 Instituto de Saúde Coletiva, Universidad Federal de Bahia, Rua Basílio da Gama, S/N, Campus Universitário Canala, Salvador, 40.110-040, Brazil; 8 Infectious Diseases Epidemiology, London School of Hygiene and Tropical Medicine, Keppel Street, London, WC1E 7HT, UK; 9 Community Health Sciences, St George's University of London, Cranmer Terrace, London, SW17 ORE, UK; 10 Centre for Infectious Diseases, St George's University of London, Cranmer Terrace, London, SW17 ORE, UK

## Abstract

**Background:**

Geohelminth infections are highly prevalent infectious diseases of childhood in many regions of the Tropics, and are associated with significant morbidity especially among pre-school and school-age children. There is growing concern that geohelminth infections, particularly exposures occurring during early life *in utero *through maternal infections or during infancy, may affect vaccine immunogenicity in populations among whom these infections are endemic. Further, the low prevalence of allergic disease in the rural Tropics has been attributed to the immune modulatory effects of these infections and there is concern that widespread use of anthelmintic treatment in high-risk groups may be associated with an increase in the prevalence of allergic diseases. Because the most widely used vaccines are administered during the first year of life and the antecedents of allergic disease are considered to occur in early childhood, the present study has been designed to investigate the impact of early exposures to geohelminths on the development of protective immunity to vaccines, allergic sensitization, and allergic disease.

**Methods/Design:**

A cohort of 2,403 neonates followed up to 8 years of age. Primary exposures are infections with geohelminth parasites during the last trimester of pregnancy and the first 2 years of life. Primary study outcomes are the development of protective immunity to common childhood vaccines (i.e. rotavirus, *Haemophilus influenzae *type B, Hepatitis B, tetanus toxoid, and oral poliovirus type 3) during the first 5 years of life, the development of eczema by 3 years of age, the development of allergen skin test reactivity at 5 years of age, and the development of asthma at 5 and 8 years of age. Potential immunological mechanisms by which geohelminth infections may affect the study outcomes will be investigated also.

**Discussion:**

The study will provide information on the potential effects of early exposures to geohelminths (during pregnancy and the first 2 years of life) on the development of vaccine immunity and allergy. The data will inform an ongoing debate of potential effects of geohelminths on child health and will contribute to policy decisions on new interventions designed to improve vaccine immunogenicity and protect against the development of allergic diseases.

**Trial registration:**

Current Controlled Trials ISRCTN41239086.

## Background

The geohelminth (also known as intestinal or soil-transmitted helminth infections) parasites, *Ascaris lumbricoides, Trichuris trichiura*, hookworm, and *Stronglyoides stercoralis*, are common infectious diseases of childhood in tropical regions and are estimated to infect over 2 billion humans worldwide [1]. Geohelminth infections are considered to cause significant morbidity in endemic areas through affects on nutrition, growth, and cognition affecting school performance [2].

Geohelminth infections induce an immune responses in humans characterized by elevated IgE levels, eosinophilia, and increased production of Th2 cytokines from peripheral blood leukocytes in response to stimulation by parasite antigen [3]. While initial exposures to these parasites may be associated with enhanced allergic inflammatory responses to the parasite, in long-term infections and with repeated exposures, the host inflammatory response becomes more tightly controlled [4,5]. Chronic infections have potent regulatory effects on anti-parasite inflammatory responses [5,6], being associated with a modulated or 'modified' Th2 responses that may facilitate parasite survival but protect the host from damaging immune-mediated disease [4]. The regulation of host immunity by chronic geohelminth infections may not just affect responses to parasite antigens but also other exogenous antigens such as the antigenic constituents of vaccines and aeroallergens. Such effects may contribute to the impaired vaccine immunogenicity [7-10] and decreased prevalence of allergic diseases [5,11] reported from the rural Tropics.

The prevalence of allergic diseases has increased over the past 40 years and has reached epidemic levels in many developed countries such as the UK, [12] where they are now the most prevalent chronic diseases of childhood. International surveys showed that the prevalence of asthma varied 20-fold between countries, ranging 1.6-36.8% [13], and a surprising finding was the high prevalence of asthma in urban centres in Latin America with prevalence rates as high as those reported from traditional high prevalence countries [13,14].

The causes of the high prevalence of allergic diseases in developed countries and the increasing prevalence in many developing countries are not known. The most widely accepted explanation is the 'hygiene hypothesis' that has attributed the increases in prevalence to a decline in infectious and microbial exposures during childhood [12,15,16]. This was initially explained in the context of the effects of early infectious exposures on the Th1/Th2 cytokine balance: a greater infectious burden induces stronger Th1 responses that counterbalanced the effects of pro-allergic Th2 responses. The observation that chronic helminth infections, that induce strong Th2 immunity, appeared to protect against allergy stimulated a re-working of the hypothesis to emphasize the role of immune regulatory mechanisms to control Th1 and Th2-mediated inflammation [17,18]. Current thinking has emphasized the importance of multiple early infectious exposures including helminths in the induction of an immune regulatory network [6,11,19] that controls inflammatory responses to both Th1 and Th2-inducing stimuli. Important mediators of immune regulation may include subsets of regulatory immune cells [4,20-23].

There is evidence from experimental animal models that intestinal and tissue helminth infections have deleterious effects on vaccine immune responses [24,25]. Further, intestinal helminth infections have been shown to either enhance [26,27] or suppress [27-29] allergic inflammatory responses in different experimental models of allergic inflammation. Studies conducted in children and adults indicate that concurrent geohelminth infections can suppress protective immune responses to both parenteral [30,31] and oral [32] vaccines and are inversely associated with the prevalence of allergen skin test reactivity [5,33] used as a marker for atopy or allergic sensitization. Geohelminth infections have also been associated with either a reduced [34] or increased prevalence [5,34] of asthma depending on the prevalence and type of geohelminth parasite present.

Because the antecedents of allergic diseases are considered to occur in early life and most vaccines are administered during the first year of life before geohelminth infections are acquired, there is interest in the potential effects of maternal geohelminth infections on infant morbidity [35], and the effects of such exposures on vaccine immune responses during infancy [31,35-37] and the development of allergic sensitization and allergic diseases [5,35,38,39]. There is evidence that maternal helminth infections bias the fetal immune response [31] an effect that persists into infancy and that may interfere with protective immunity associated with vaccines such as BCG [40]. Similarly, maternal ascariasis is associated with sensitization to *A. lumbricoides *antigens in newborns [41], although the long-term consequences of such sensitization are unclear.

The present study is investigating the potential effects of intrauterine and postnatal exposures to geohelminths on vaccine immune responses in infancy, and the development of allergic sensitization and allergic inflammatory diseases in childhood. The study will also investigate the immunological mechanisms by which such effects occur. The findings of this study have clear policy implications given that anthelmintic treatment of school-age children for the control of geohelminth infections is now a widely implemented public health strategy [42,43], and the use of anthelmintic treatment during pregnancy has been advocated to improve the health of mothers and infants [43,44]. Few opportunities, therefore, still exist to study the development of allergic disease in early childhood in the context of endemic geohelminth infections.

### Hypotheses

This study has been designed to investigate four specific hypotheses relating to the effect of maternal and infant infections with geohelminths on host immune responses and development of allergic inflammatory diseases: 1) chronic exposures to geohelminth infections (i.e. maternal geohelminth infections and infant geohelminth infections within the first 2 years of life) suppress immune responses to childhood vaccines; 2) chronic exposures to geohelminth infections suppress aeroallergen skin test reactivity; 3) chronic exposures to geohelminth infections protects against the development of eczema; and 4) chronic exposures to geohelminth infections protect against the development of asthma but non-chronic exposures (i.e. later childhood exposures occurring after 2 years in the absence of maternal geohelminth infections) increase the risk of asthma.

## Methods/design

### Study design

**Figure 1:**
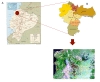
**Study site**. A. Map of Ecuador showing location of District of Quinindé, Esmeraldas Province (black oval) (Courtesy of The General Libraries, The University of Texas at Austin). The recruitment area for the cohort was defined by the geographic boundaries of this district. B. Map showing parishes the District of Quinindé including La Concordia. H-Hospital Padre Alberto Buffoni. C. Geographic location of households of cohort infants.

The study is a birth cohort of 2,403 children recruited from around the time of birth in HPAB over the period November 2005 to December 2009. The recruitment area for the cohort is defined geographically by the boundaries of the District of Quinindé, Esmeraldas Province in Northern coastal Ecuador (Figure 1). The primary exposures are maternal and infant infections with geohelminths and the primary outcomes are vaccine immunity, atopy and allergic disease.

### Study population and area

The Province is one of the poorest regions of Ecuador, with a per capita income of less than US$2,000 in 2005. Quinindé is a rural District that covers an area of 3,471 km^2 ^and includes 1 urban (the town of Quinindé) and 6 rural parishes with an estimated population of 150,000. The District is located in an area of (formerly) equatorial rainforest that has largely been cleared, at an altitude of approximately 100 m and average annual day temperature of 30°C and 75% humidity. The District has an ethnically mixed population of mestizos (90%), Afro-Ecuadorians (7%), and Amerindians (3%). Twenty-two percent of the population is estimated to be urban (Quinindé town) and 78% rural. In the town of Quinindé, approximately 90% of the population have access to electricity, 60% to treated drinking water, 40% to sanitation; 60% to solid waste disposal services. In contrast, in the rural areas, 10% have access to electricity and none have access to other services. The main sources of income are derived from African palm oil and fruit cultivation, cattle, and extraction of timber. The study is based at the Hospital "Padre Alberto Buffoni" (HPAB) (H in Figure 1) in Quinindé town. HPAB is the only Hospital serving the District and the only health centre with maternity services.

### Inclusion criteria

Although assessments and sampling are conducted during pregnancy, only a proportion of pregnant mothers attend antenatal clinics, and formal recruitment into the cohort occurs around the time of birth. Entry criteria into the study are: 1) healthy normal baby less than 14 days old; 2) at least one stool sample collected from the mother; 3) the family has lived in the District for the last 2 years and does not plan to move out of the District over the following 3 years; 4) the home is accessible; and 5) the mother is 17 years or older.

### Sampling and evaluations

The sampling and examination schedule for follow-up is shown in Table 1. Evaluations are conducted at birth, 2 weeks, 3, 7, 13, 18, 24 and 30 months, and at 3, 5, and 8 years through home visits and scheduled visits at the ECUAVIDA outpatient clinic at HPAB. More detailed immunologic evaluations are performed in an immunology sub-cohort (ISC) of the last 295 infants recruited using blood samples collected at birth, 7, 13 and 24 months, and at 3, 5 and 8 years. A surveillance sub-cohort of 195 newborns living within the town of Quinindé are being followed-up actively and sampled for viral respiratory tract infections (influenza viruses, respiratory syncytial viruses, adenoviruses, and rhinoviruses) using nasal swabs and diarrheal illnesses (rotavirus and norovirus) using stool samples during the first 2 years of life. Aliquots of all stool samples collected and hypopharyngeal swabs (at routine sampling times) are being stored for future analyses of bacterial microbiota.

**Table 1:** Sampling schedule for ECUAVIDA cohort

Visit	Ante-natal	Birth	< 2 wks	3 mths	7 mths	13 mths	24 mths	3 years	5 yrs	8 yrs
**Site**	**HPAB**	**HPAB**	**Home**	**HPAB/home**	**HPAB/home**	**Home**	**HPAB/home**	**HPAB/home**	**HPAB/home**	**Home**

Child										
Questionnaire		X	X		X	X	X	X	X	X
Faeces		X	X	X	X	X	X	X	X	X
Blood		X^a^	X		X	X	X	X	X	X
Clinical exam		X	X		X	X	X	X	X	X
Anthropometry		X			X		X	X	X	X
SPT							X	X	X	X
PFTs/BHR										X
Mantoux test										X

Mother										
Questionnaire	X	X	X	X						
Faeces	X	X	X							
Blood	X									
SPT										

Father										
Faeces			X	X						
SPT										

Household										
GPS			X^b^			X^b^				
Dust			X^b^							
Faeces			X							

Stool samples are collected also at 18 and 30 months. HPAB-Hospital Padre Alberto Buffoni in Quinindé, Esmeraldas Province; SPT-allergen skin test reactivity; PFTs-pulmonary function tests measured by spirometry; BHR-bronchial hyperresponsiveness or evidence of reversibility following β_2 _agonist; GPS-geographic positioning system measurements for mapping of households. ^a ^immunology sub-cohort only. ^b ^measurements repeated for change of address.

### Vaccination schedule

All vaccines are provided free of charge by the Ministry of Public Health at the vaccination clinic at HPAB. The vaccination schedule is: birth-BCG; 2 months and 4 months-pentavalent (Quinvaxem [DPT-HepB-Hib], Novartis), trivalent oral poliovirus vaccine (Chiron), rotavirus (Rotarix, GSK); 6 months-pentavalent, OPV; 12 months-measles-mumps-rubella (Serum Institute of India [SII]); and 18 months-DT (SII) and OPV.

### Measurement and definition of geohelminth exposures

Stool samples to measure geohelminth infections have been collected from mothers in the third trimester of pregnancy (stool samples collected after birth and before the child is 14 days old are considered to represent a third trimester stool sample where the mother has not received anthelmintic treatment) and from infants at 3, 7, 13, 18, 24, and 30 months, and at 3, 5, and 8 years. Stool samples are analysed using a combination of standard methods including direct saline mounts, the modified Kato-Katz method, formol-ether concentration, and carbon-coproculture methods [45]. Geohelminth infectious exposures will be defined as follows: 1) maternal geohelminth infections-presence of any geohelminth infection detected during the 3^rd ^trimester of pregnancy; and 2) infant geohelminth infections-presence of any geohelminth infections detected during the first 2 years of life.

### Measurement and definitions of study outcomes

#### Primary study outcomes

1. *Vaccine immune responses: *vaccine immunity will be measured by the presence of protective antibody levels measured as follows-1) IgG to tetanus toxoid as described previously [46]. Immune protection will be defined > 0.15 IU/ml; 2) titer of neutralizing antibodies to OPV type 3 using the microneutralization assay at the Health Protection Agency (HPA), London, UK. Protection will be defined as OPV type 3 virus neutralization at 100TCID50 at serum dilution ≥ 1:8 dilution; 3) IgA to rotavirus (HPA); protection indicated by titers ≥ 20 U/mL; 4) IgG to polyribosylribitol phosphate of *Haemophilus influenzae *type B (Binding site, Birmingham, UK); protection > 0.15 mg/mL; 5) anti-HBS IgG antibodies (Architect, Abbott Diagnostics, Sligo, Ireland), protection > 10 IU/mL IgG. Plasma samples collected at 24 months and 5 years will be used to measure short-term (24 months) and long-term (5 years) antibody-mediated protective immunity. Vaccine responses to Hib and rotavirus will be measured only at 7 months to distinguish vaccine-mediated responses from those acquired from natural exposures to these infections.

2. *Atopy: *defined by the presence of allergen skin test reactivity at 5 years to any of the following aeroallergens: *Dermatophagoides pteronyssinus/farinae *mix, American cockroach (*Periplaneta americana*), fungi mix, dog, cat, and mixed grass pollen. A positive test is defined by the presence of a skin wheal ≥ 3 mm greater than saline control. All skin testing will be conducted by trained physicians and will be supervised by a highly experienced clinical investigator (MEC).

3. *Eczema: *Eczema has been assessed by questionnaire and physical examination using standardized instruments based on the United Kingdom Working Party (UKWP) criteria/Nottingham protocol [47]. Cases of flexural dermatitis are assessed further for severity using the SCORAD protocol [48]. Evaluations for eczema are performed at 7, 13, 24, and 36 months. Infants presenting to the ECUAVIDA outpatient clinic with skin complaints are also assessed for eczema. All clinical evaluations are done by trained physicians using standardized protocols. Eczema will be defined by at least one presentation during the first 3 years of life of an itchy skin condition plus 3 or more of the following: i) history of involvement of flexural sites including cheeks; ii) history of atopic disease in first degree relative: iii) a history of generally dry skin in last year; and iv) visible flexural dermatitis or dermatitis affecting cheeks/forehead and outer limbs.

4. *Asthma: *Asthma will be measured at 5 and 8 years using the ISAAC phase II questionnaire [49] and defined by the presence of wheeze within the previous 12 months plus: either a previous history of wheeze within the previous 12 months (at 13, 24, and 36 months) or a previous ECUAVIDA clinic diagnosis of asthma. Non-atopic and atopic asthma will be defined by the presence or absence, respectively, of allergen skin test reactivity. Measurement of pulmonary function and reversibility with a short-acting β_2_-agonist will be done at 8 years of age. Wheezing illness will be defined as wheeze in the previous 12 months.

### Secondary study outcomes-immunological outcomes

Five possible mechanisms have been defined that could mediate the putative effects of geohelminth infections on the primary study outcomes. A justification for choosing these mechanisms is provided in the Discussion. The mechanisms are: 1) Th2 polarization-measured by the ratio of IL-5 to IFN-γ protein produced by peripheral blood leukocytes (PBLs); 2) immune homeostasis-production of IL-10 spontaneously in 5-day PBL cultures [50]; 3) immune suppression-antigen-specific suppression measured by frequencies of IL-10^+^CD4^+ ^T cells or IL-10 production by antigen-stimulated PBLs [51-53] or 'bystander suppression' measured by IL-10 production by *A. lumbricoides *antigen-stimulated PBLs [54,55]; 4) immune maturation-capacity of PBLs to produce IFN-γ to sub-optimal stimulus with Staphylococcal enterotoxin B (SEB); and 5) pro-inflammatory responses-IL-8 production by PBLs stimulated with LPS [56], and quantities of IL-17, IFN-γ, and IL-5 produced by SEB-stimulated PBLs. Flow cytometry experiments will be performed using cryopreserved and fixed peripheral blood leukocytes using standardized protocols [41,56,57].

### For each of the outcomes, the following mechanisms will be evaluated

1. *Vaccine immunity*: Immunological outcomes will be measured at 24 months (short-term immunity) and 5 years (long-term immunity) for Th2 polarization (tetanus toxoid [TT] and tuberculin [PPD]-stimulated PBLs), immune homeostasis, and immune suppression (IL-10 production by PBLs stimulated with TT, PPD, and *A. lumbricoides *antigen).

2. *Atopy*: Immunological outcomes will be measured at 5 years for immune homeostasis and immune suppression (frequencies of IL-10+CD4+ T cells [ISC only] or IL-10 production by PBLs stimulated with *D. pteronyssinus, P. americana*, and *A. lumbricoides *antigen).

3. *Eczema: *Immunological outcomes will be measured up to 3 years for markers of immune maturation, immune homeostasis, immune suppression (frequencies of IL-10^+^CD4^+ ^T cells (ISC only) or IL-10 production by *A. lumbricoides*-stimulated PBLs), and pro-inflammatory responses (ISC only).

4. *Asthma*: Immunological outcomes will be measured at 5 years for markers of immune homeostasis, immune regulation/suppression (frequencies of IL-10^+^CD4^+ ^T cells [ISC only] and IL-10 production by PBLs stimulated with *D. pteronyssinus *and *A. lumbricoides *antigen), Th2 polarization (ratio of IL-5 to IFN-γ to sub-optimal SEB stimulus), and pro-inflammatory responses (ISC only).

### Study limitations

We expect losses to follow-up to be up to 10% in the first year (i.e. 2,163/2,403 followed up) and 5% annually to age 3 and a further 5% between ages 3 and 5. Estimates for the number of children followed-up are: 2 years-2,055, 3 years-1,952, and 5 years-1,854. Losses are due to the mobility of the study population.

Potential sources of biases are selection bias caused by losses to follow up and information bias caused by systematic misclassification of outcomes. The following methods are being used to minimize these biases: *Achieving high rates of follow-up *- a) we have a well-trained study team employed full-time for the cohort; b) we conduct regular home visits to maintain contact with study mothers; c) the universal use of mobile phones allows us to maintain regular contact with study mothers; d) the mother of each child is given a cohort identification card with a phone number to make appointments at the ECUAVIDA outpatient Clinic; e) changes of address are followed up actively with home visits to repeat environmental sampling. *Information bias *- a) repeated measurements of outcomes (for asthma, eczema, and allergen skin test reactivity) should reduce misclassification and recall bias; b) clear definitions for primary outcomes and exposures; c) evaluation of outcomes is being performed blind to exposure status. Detailed information on potential confounding factors is being collected.

### Study power

The study sample size is fixed at 2,403 newborns. Primary outcome variables will be treated as statistically independent. Power calculations for primary study questions are shown in Table 2[58,59]. We expect a high degree of power for the estimation of most primary and secondary study outcomes (≥ 80% depending on immunological mechanism at α = 0.01) that will allow for the loss of power expected by controlling for confounding.

**Table 2:** Power for analysis of primary exposure-outcome associations

Primary exposures (geohelminth infections)	Outcome	Sample size available	Analysis sample size	Exposure prevalence	Expected prevalence of outcomes (exposed vs. unexposed groups)	Α	Power
Maternal	Vaccine antibodies @ 7 mths						
	Hib	2,300	1,000	50%	65% vs. 75%	0.01	80%
	Rotavirus	2,300	1,000	50%	40% vs. 60%	0.01	> 99%
Maternal	Vaccine antibodies @ 2 yrs						
	TT	1,955	1,000	50%	85% vs. 95%	0.01	> 99%
	HBV	1,955	1,750	50%	80% vs. 85%	0.05	80%
	OPV type 3	1,955	500	50%	80% vs. 95%	0.01	> 99%
Infant	Vaccine antibodies @ 5 yrs						
	TT	1,725	1,000	35%	60% vs. 80%	0.01	99%
	HBV	1,725	1,500	35%	70% vs. 80%	0.01	95%
	OPV type 3	1,725	1,000	35%	50% vs. 70%	0.01	> 99%

Maternal	SPT @ 3 years	1,840	1,840	50%	15% vs. 25%	0.01	99%
Infant	SPT @ 3 years	1,840	1,840	35%	15% vs. 25%	0.01	> 99%

Maternal	Eczema @ 3 years	1,840	1,840	50%	25% vs. 35%	0.01	99%

Maternal	Asthma @ 5 years	1,725	1,725	50%	17% vs. 23% #	0.05	86%
Infant	Asthma @ 5 years	1,725	1,725	35%	17% vs. 23% #	0.05	83%
Infant	Asthma @ 5 years	1,725	1,207	20%	25% vs. 15% #	0.01	93%

Random samples of the study population will be selected for analysis of the protective levels of vaccine antibodies. Based on data from the cohort we estimate that 20% of children will have allergen skin test reactivity to any allergen at 5 years, that 30% of children will have at least one documented episode of eczema by 3 years of age and that 20% of children will have asthma at 5 years. Based on data from the cohort we estimate that ~50% of mothers are infected with any geohelminth parasite and ~30% of children will have at least one documented geohelminth infection during the first 2 years of life.

#-expected effects of geohelminth infections on asthma prevalence using data from cross-sectional analyses in Ecuador [58] and Brazil [59].

### Analysis plan

**Figure 2:**
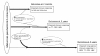
**Conceptual model for effects of maternal and infant geohelminth infections on vaccine immune responses measured at 7 months, 2 years or 5 years**. Maternal infections would be expected to mediate effects on vaccine responses at 7 months and 2 years, although infant infections, generally acquired during the second year of life, may contribute to effects at 2 years. The suppressive effects of infant infections would be expected to be most important at 5 years. Potential immunological mechanisms that may mediate the suppressive effects of geohelminth exposures are shown in dashed boxes.

**Figure 3:**
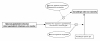
**Conceptual model of effects of maternal and infant geohelminth infections on allergen skin test reactivity**. The effects of both exposures are presumed to occur via the development of a modified Th2 (mTh2) response during chronic geohelminth infection. A modified Th2 response may affect allergen skin test reactivity through 2 distinct mechanisms: 1) a direct effect on mast cell function independent of aeroallergen-specific IgE resulting in an increased threshold for activation through enhanced production of spontaneous or geohelminth-induced IL-10. Such effects would be predicted to effect wheal sizes non-specifically including the histamine positive control. 2) An indirect effect on mast cell function by attenuation of the association between specific IgE and skin test reactivity. This effect would be predicted to be aeroallergen-specific, affect only those wheals for which an individual has significant levels of specific IgE (at > 0.35 kU/L) but not the positive control, and be mediated by aeroallergen-induced IL-10. The effect could also be mediated by geohelminth-induced IL-10 for aeroallergens where the IL-10-stimulating allergens share significant immunological cross-reactivity with helminth allergens.

**Figure 4:**
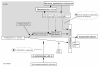
**Conceptual model for the effects of maternal and infant geohelminth infections on asthma in children**. The effects of these exposures on asthma are considered to occur via effects on bronchial hyperreactivity. The protective effects of chronic helminth exposures (maternal and infant infections within the first 2 years of life) are presumed to occur via the development of a modified Th2 (mTh2) response that may affect bronchial hyperreactivity through: 1) Immune regulation/suppression-suppression of airways inflammation via enhanced production of geohelminth or aeroallergen-induced IL-10. 2) Homeostasis-enhanced production of spontaneous IL-10 will suppress airways inflammation non-specifically. Alternatively children without early geohelminth exposures and that are first exposed to infection after 2 years of age (> 24 m) may respond to *A. lumbricoides *infection with strong inflammatory responses in the airways that up-regulate pro-inflammatory pathways including Th1, Th2, and Th17. The modified Th2 response will be defined by the presence of specific IgG4 antibodies to geohelminths. RTIs- respiratory tract infections. ETS-environmental tobacco smoke.

**Figure 5:**
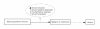
**Conceptual model for the effects of maternal geohelminth infections on eczema**. Effects of maternal geohelminth exposures on eczema are considered to occur through the capacity of the immune system to regulate inflammation of the skin caused by external insults. Factors that may determine the ability of the immune system to do this include: 1) Immune maturation-the speed of maturation of the immune response in early life is likely to effect the ability of the immune response to respond to infections and other insults with appropriate but measured inflammatory responses. 2). Immune regulation/suppression-the development of robust and specific immune regulatory mechanisms are important for the development of an appropriate inflammatory response to specific insults. 3) Immune homeostasis-robust mechanisms to limit responses to pro-inflammatory stimuli may be an importance mechanism for the control of inflammation.

Statistical analysis will be guided by the conceptual frameworks presented for each of the study outcomes (Figures 2, 3, 4 and 5). The analysis of the primary study outcomes will use multiple logistic regression to estimate the Odds Ratios for exposure-outcome associations with adjustment for appropriate confounding factors. For example, *a priori *confounders for the association between maternal geohelminth infections and eczema by 3 years of age are gender, maternal educational level, household crowding, and infant geohelminth infections. The primary analyses will evaluate the effects of maternal geohelminth infections or infant geohelminth infections on: 1) protective immunity to common childhood vaccines: 2) the presence of allergen skin test reactivity at 5 years of age; 3) the development of eczema by 3 years of age; and 4) the development of asthma at 5 and 8 years of age. Immunological variables will be defined as binary variables with a cut-off defined by the limit of detection of the assays (i.e. IL-10) or median level (i.e. IL-8) or as log_e_-transformed continuous variables. Because immunological variables are considered as intermediate in the causal pathways between exposures and outcomes, these variables will be evaluated in hierarchical analyses [60].

### Information, ethical approval, consent, and ethical considerations

The Ministry of Health in Ecuador was informed of the study and gave its support for the study in Esmeraldas Province, Ecuador (Subsecretaria General de Salud, reference SSG-10-000285). Formal recruitment into study occurred at the first home visit done during before the child was 14 days old. Informed consent for the collection of samples during the study before this time was done at 2 different time points: 1) antenatal clinic visit for collection of a stool and blood samples: 2) in the maternity department of HPAB for collection of maternal stool and blood samples, cord blood and meconium. At each of the three times at which written consent was obtained, the mother received both written and verbal information in Spanish on the aims of the study and the reasons for collection of study samples. Results are provided to the mother for all clinical samples collected. Where necessary, the child will be evaluated by a study physician, and appropriate medication and nutritional supplements provided as required through the ECUAVIDA study clinic at HPAB. Appropriate antiparasite treatment is given for all stool samples with documented geohelminth infections. Benzimidazole drugs are not given to pregnant women or children before 2 years of age following the recommendations of the Ecuadorian Ministry of Health. Infected mothers are offered albendazole when breast-feeding has finished. Infected children aged below 2 years are treated with pyrantel and after 2 years with albendazole. Infections with *Giardia intestinalis *and *Entamoeba histolytica *are treated with a standard course of metronidazole if characteristic trophozoites are observed. The study protocol was approved by the Ethics Committees of the Hospital Pedro Vicente Maldonado and the Universidad San Francisco de Quito, and is registered as an observational study (ISRCTN 41239086).

Although anthelmintic treatment is a highly popular intervention, clear health benefits from the treatment of pregnant women have not been unequivocally demonstrated, and such benefits could be argued to be at best marginal in most endemic regions where hookworm anaemia is relatively infrequent and geohelminth infections are low intensity [61]. For this reason, we opted for an observational rather than an interventional design that would allow us to understand the natural history of the interaction between early geohelminth exposures and the study outcomes, but provide the opportunity to identify potential interventions that could be beneficial and be evaluated in future intervention studies.

## Discussion

The ECUAVIDA cohort study has been designed to investigate the potential impact of early exposures to geohelminth infections during pregnancy and in the first 2 years of life on protective immunity provided by widely used infant vaccinations and on the development of allergic sensitization and allergic diseases. The present study is one of several observational and intervention studies [18,35] currently in progress that will inform public health policy of the potential effects of early exposures to these parasites on a number of health outcomes and the potential benefits as well as risks of providing anthelmintic drugs during pregnancy and infancy in areas where these parasites remain highly prevalent. Adequate evidence to support changes in public health policy will require studies to be conducted in different geographic settings and among different populations with varying prevalence of geohelminth parasites.

### Potential effects of geohelminth infections on vaccine immune responses

Currently, the most effective public health intervention aimed at reducing infectious disease deaths in under-5s is infant vaccination. Several vaccines are recognized to be less immunogenic in poor populations [7,8,10,62-66] requiring an increase in the dose or number of doses administered to achieve adequate vaccine immunity [7,67]. Poor vaccine immunogenicity is a problem for mucosal vaccines such as OPV and rotavirus both of which have proved to be less immunogenic in poor compared to wealthier populations [9,10,68,69]. In the case of OPV, poor vaccine immunity is most marked to OPV type 3 [7,70,71], and as many as 11 doses of OPV may be required to achieve 90% seroconversion [72]. Factors contributing to poor responses to vaccines may include overcrowding, poor sanitation, high-titer maternal antibodies, micronutrient malnutrition, environmental enteropathy and co-infections [8,73,74]. The relative importance of these factors in affecting vaccine immune responses may vary between vaccines.

Geohelminth infections have deleterious effects on vaccine immunity to oral [32,75] and parenteral vaccines [30,31] in children, and there is growing evidence from experimental animal models that concurrent parasite infections have important suppressive effects on vaccine immune responses [25,76,77]. Most human studies to date have been conducted in school age children or adults. There are few data from studies investigating the effects of maternal geohelminth infections on vaccine immune responses in infancy. Such studies have evaluated effects of maternal helminth infections and have provided evidence for a suppressive effect of maternal infection on IFN-γ responses to mycobacterial antigens in newborns [40] and IgG antibody responses to *Haemophilus influenzae *type b vaccine (Hib) at 6 months of age [31].

The immunological mechanisms by which such infections mediate these effects are poorly understood. Geohelminth exposures may suppress vaccine immune responses through three possible immunological mechanisms (Figure 2): 1) Th2 polarization-with a switch in Th1 towards Th2 responsiveness causing reduced Th1 responses (i.e. IFN-γ) responses important for antiviral immunity and immunity against bacterial infections (e.g. *Mycobacteria *[78]*and Bordetella pertussis *[79]). Such a mechanism would predict an overall and non-specific Th2-biassed response to both vaccine and other antigens with a failure to develop 'protective' Th1 vaccine responses. 2) Immune homeostasis-attenuated immune responses caused by strong homeostatic mechanisms such as increased spontaneous production of IL-10 associated with chronic geohelminth infections [80]. Such effects would be expected to suppress Th1 and Th2 immune responses to vaccines and may result in an increased threshold for the activation of immune responses. 3) Immune suppression-this could be antigen-specific or caused by bystander suppression. Previous studies have demonstrated that the suppression of immunity to TT in vaccinated subjects infected with *Onchocerca volvulus *[51] and *Wuchereria bancrofti *[52] is mediated in part by TT-specific IL-10. Alternatively, strong regulatory responses to helminth antigens associated with enhanced geohelminth antigen-induced IL-10 [80] may inhibit immune responses to unrelated vaccine antigens through bystander suppression.

### Potential effects of geohelminth infections on allergic sensitization

Childhood geohelminth infections are inversely associated with allergen skin test reactivity [81-87]. This observation has led to the suggestion that anti-parasite immune modulatory mechanisms may suppress allergic inflammatory responses to aeroallergens. However, the findings of intervention studies in which children infected with geohelminths have received anthelmintic treatment are contradictory. Different intervention studies have provided evidence for an increased incidence [88] or prevalence [55,89,90] of allergen skin test reactivity after periodic anthelmintic treatments, but a large cluster-randomized trial of periodic anthelmintic treatment in Ecuador did not observe a change in the prevalence of allergen skin test reactivity after treatment [91]. The contradictory findings of different studies could be explained by differences in the prevalence of geohelminth parasites between different geographic regions, the period for which anthelmintic treatment was given, the effects of early exposures to geohelminths in programming anti-allergic responses [5,39], or treatment effects on other endemic helminth infections (e.g. *Toxocara*).

The presence of geohelminths during pregnancy may modify the immune responses of offspring *in utero *[41]. The possible protective effects of environmental exposures such as geohelminths against allergy may be strongest when they occur in the first year or two of life [92]. We have hypothesized that early exposures to geohelminths may programme the infant immune system for reduced allergy [39]. Such programming may not be reversible (i.e. by anthelmintic treatment in later childhood) and could be 'reinforced' later by active regulatory mechanisms induced by heavy helminth infections (reversible by anthelmintic treatment) [39]. Evidence in support of this hypothesis has come from a recent observational study in Brazil showing that early and heavy exposures to *T. trichiura *protected against allergen skin test reactivity later in childhood independent of later infections [93].

The immunological mechanisms by which chronic geohelminth infections may suppress IgE-mediated hypersensitivity measured by allergen skin test reactivity are illustrated in Figure 3. Maternal geohelminth infections, augmented by infections in infancy, may have indirect suppressive effects on immediate hypersensitivity responses in the skin through effects on immune homeostasis. Chronic geohelminth infections in children are associated with an increased accumulation in vitro by unstimulated PBLs of the anti-inflammatory cytokine IL-10 [50]. A previous study of children from Cameroon infected with *A. lumbricoides *and *T. trichiura *provided evidence for an inverse association between geohelminth infection intensity and immune reactivity that was associated with enhanced production *in vitro *of IL-10 and TGF- β1 by unstimulated PBLs [94]. Further, a study of children in poor neighborhoods in Salvador, Brazil has shown that the presence of detectable spontaneous IL-10 is associated with poor access to sanitation [95] and geohelminth infections in early childhood [80]. Elevated 'homeostatic' production of IL-10 by immune cells may suppress immune reactivity [5]. IL-10 has been shown previously to suppress mast cell activation [96] and be inversely associated with wheal size to allergen skin tests [97].

In populations living in environments endemic for geohelminth infections, there is a strong disassociation between the presence of allergen-specific IgE in serum and skin test reactivity to the same allergen [57,82,98,99]. Environmental exposures including geohelminth infections are likely to be major determinants of this relationship. Geohelminth infections may affect the association between allergen-specific IgE in serum and skin test reactivity through enhanced production of helminth antigen-induced [54], aeroallergen-induced [53], or spontaneous IL-10 [50]. Increased IL-10, whatever the cellular source, may suppress mast cell activation in the skin, thereby reducing the prevalence of allergen skin test reactivity. The effects of maternal geohelminth or early infant infections on levels of allergen-specific IgE are not clear. A high proportion of children living in the rural Tropics appear to have low-affinity IgE to aeroallergens (e.g. 0.35-0.7 kU/L) that can be only be partially absorbed by pre-incubation with the specific allergen [98] and may represent cross-reactive anti-helminth IgE antibodies [100,101].

### Potential effects of geohelminth infections on the development of allergic inflammatory diseases

*Asthma *is a heterogeneous disease caused by complex interactions between host genetics and environment. Rural residence appears to be strongly protective against the development of allergic disease that may be mediated by common exposures present in the rural environment [102], particularly in early life [90]. Early infections are considered to be particularly relevant [92,103-107]. Geohelminth infections in childhood are inversely associated with asthma symptoms [34] or specifically asthma associated with atopy [87] in some populations that are highly endemic for these parasites. The effects of different geohelminths parasites on asthma may be distinct [34]. However, there is also growing literature that points to a role of helminths, particularly *A. lumbricoides*, as a risk factor for asthma in populations with a low prevalence of infection [108-114].

Atopy has been consistently linked to asthma in developed countries but there is evidence that the association between asthma and atopy in developing countries is weaker [87,98,115-117]. An explanation for this observation is that is that the more prevalent infectious exposures including geohelminth infections present in many developing countries may attenuate the association through a reduction in allergic sensitization.

The mechanisms by which geohelminth infections may affect inflammatory disease of the airways are illustrated in Figure 4. A predisposition to inflammation of airways and the pathological sequelae (e.g. airways remodeling) may arise from an interaction between genetic and undefined early life *in utero *exposures [118]. The subsequent development of asthma may be associated with or without atopy (so-called atopic and non-atopic asthma). Because chronic geohelminth infections have important modulatory effects on allergic inflammation [5], it is likely that protective exposures mediate their effects by suppression of allergic inflammation through the 'atopic' pathway (Figure 4). The mechanisms that mediate this effect are unlikely to be restricted to the lungs-experimental animal models of the effects of intestinal helminths on tissue inflammation have shown that infections can ameliorate inflammatory disease in several different tissues (e.g. the intestine, liver, and lungs [4,119-121]. Relevant mechanisms are likely to be generalized and may include effects on immune homeostasis (i.e. increased spontaneous IL-10) and regulation (i.e. elevated frequencies of adaptive T regulatory cells expressing IL-10 [Tr1 cells]). Migratory *Ascaris *larvae in populations where *A. lumbricoides *infections are sporadic or low-prevalence populations may induce inflammation directly in the lungs or enhance other inflammatory processes (e.g. inflammation caused by exposure to environmental tobacco smoke or respiratory tract infections: Figure 4).

*Eczema *is a chronic relapsing inflammatory skin disease that may be caused by an impaired ability to regulate inflammation induced by environmental insults in the presence of impaired skin barrier function [122]. Eczema generally develops within the first 6 months of life [123], peaks in prevalence between 2 and 3 years [124], after which the prevalence declines [125]. Little is known of the natural history of eczema in tropical environments with respect to prevalence, severity, environmental risk factors, and associations with atopy. The association between eczema and geohelminth infection is controversial [126] and studies have shown both positive [127] and negative [128-130] associations. An intervention study in Uganda provided evidence that treatment of geohelminths during pregnancy was associated with a reduced risk of eczema at 1 year [130]. Infants of infected mothers in the placebo group had a reduced prevalence of eczema compared to those of non-infected mothers [130].

The mechanisms by which geohelminth infections may reduce the risk of eczema are not known. Possible mechanisms are illustrated in Figure 5. The strongest effects are likely to be mediated by maternal infections because the disease often appears before infant geohelminth infections are acquired. The clinical presentation of eczema is a direct consequence of the damage caused by repeated scratching of the skin. Eczema, may therefore, be caused by numerous external insults that cause pruritus. A novel finding is that children born in the rural Tropics rapidly down-regulate pro-inflammatory responses to innate immune stimuli [56]. Such age-dependent responses are likely to be important adaptive mechanisms by which an early capacity to respond to innate immune stimuli that may be important in the absence of adaptive immunity but associated with an increased risk of pathologic inflammation, is rapidly down-regulated once specific adaptive immune responses have developed. We hypothesize, therefore, that the protective effects of maternal geohelminth infections are likely to be associated with markers of early immune maturation and mediated by non-specific and generalized anti-inflammatory effects (e.g. increased spontaneous IL-10) and regulation of innate (i.e. reduced IL-8 production to TLR agonists) and adaptive (i.e. elevated frequencies of induced regulatory T cells [i.e. CD4^+^IL-10^+ ^Tr1 cells]) immunity.

## Conclusion

The present study will investigate the possible effects of maternal and infant infections with geohelminth parasites on vaccine immunity, and the development of atopy and the allergic diseases, eczema and asthma. A greater understanding of the impact of geohelminth exposures on vaccine immune responses is likely to lead to the development of new interventions designed at enhancing vaccine immunogenicity among neglected populations where these infections are endemic especially since these populations bear a disproportionate burden of vaccine-preventable infectious diseases. Although eczema and asthma are major public health problem of childhood worldwide, relatively little is known of causal immunological mechanisms in tropical populations where the predominant disease phenotypes (e.g. non-atopic asthma) may be distinct. The present study will collect epidemiological and immunological data prospectively from birth to understand how early geohelminth exposures alter inflammatory and regulatory responses to affect the development of these inflammatory diseases. Results of this study will inform prevention strategies that could be designed to mimic the beneficial immunological effects of geohelminth infection in the absence of actual infection.

## List of abbreviations

DPT: Diptheria-Pertussis-tetanus toxoid vaccine; DT: Diptheria-tetanus toxoid vaccine; ECUAVIDA: Estudio eCUAtoriano del impacto de infecciones sobre Vacunas, Inmunidad y el Desarollo de enferemedades Alergicas; HPA: Health Protection Agency, Colindale, London, UK; HPAB: Hospital Padre Alberto Buffoni, Quinindé, Esmeraldas Province, Ecuador; HepB: Hepatitis B; Hib: ***Haemophilus influenzae ***type B; IgE: immunoglobulin E; IL- interleukin; ISAAC: International Study of Asthma and Allergies in Childhood; OPV: oral poliovirus vaccine; PBLs: peripheral blood leukocytes; PPD: purified protein derivative or tuberculin; SCAALA: Social Changes, Asthma, and Allergies in Latin America; SCORAD: SCORing Atopic Dermatitis; SEB: *Staphyloccus aureus *enterotoxin B; TT: tetanus toxoid.

## Competing interests

The authors declare that they have no competing interests.

## Authors' contributions

PJC had the original idea for the study, designed the study, has overall responsibility for the study, and drafted the manuscript. MC was involved in the design of the study and is coordinating the conduct of the study in Quinindé. CS is assisting in the co-ordination of the study in Quinindé and is supervising the clinical laboratory in Quinindé. FG is the Director of the Hospital Padre Alberto Buffoni and is assisting in the conduct of the study. EM is assisting with analysis of study samples by flow cytometry. TAEPM is advising on clinical allergy and measurement of IgE. MLB, LCR, DPS, and GEG advised on study design and are assisting with methodological issues and statistical analyses. All authors read and approved the final manuscript.

## Pre-publication history

The pre-publication history for this paper can be accessed here:

http://www.biomedcentral.com/1471-2334/11/184/prepub
